# Genomic insights into *Staphylococcus equorum* KS1039 as a potential starter culture for the fermentation of high-salt foods

**DOI:** 10.1186/s12864-018-4532-1

**Published:** 2018-02-13

**Authors:** Jong-Hoon Lee, Sojeong Heo, Do-Won Jeong

**Affiliations:** 1 Department of Food Science and Biotechnology, Kyonggi University, Suwon, 16227 Republic of Korea; 2 Department of Food and Nutrition, Dongduk Women’s University, Seoul, 02748 Republic of Korea

**Keywords:** *Staphylococcus equorum*, Fermentation, Jeotgal, Starter, Bacteriocin, CRISPR/Cas system

## Abstract

**Background:**

Our previous comparative genomic analysis of *Staphylococcus equorum* KS1039 with five *S. equorum* strains illuminated the genomic basis of its safety and salt tolerance. However, a comprehensive picture of the cellular components and metabolic pathways involved in the degradation of macromolecules and development of sensory properties has not been obtained for *S. equorum*. Therefore, in this study, we examined the general metabolism of *S. equorum* based on information obtained from published complete genome sequences of six *S. equorum* strains isolated from different niches. Additionally, the utility of strain KS1039 as a starter culture for high-salt food fermentations was examined.

**Results:**

All six *S. equorum* strains contained genes involved in glycolysis, the tricarboxylic acid cycle, and amino acid metabolic pathways, as well as color development. Moreover, the strains had the potential to produce acetoin, butanediol, and branched chain fatty acids, all of which are important flavor compounds. None of the strains contained decarboxylase genes, which are required for histamine and tyramine production. Strain KS1039 contained bacteriocin and CRISPR/Cas gene clusters, and experimental results suggested that these genes were functional in vitro.

**Conclusions:**

The comparative genomic analysis carried out herein provides important information on the usefulness of *S. equorum* KS1039 as a starter culture for the fermentation of high-salt foods in terms of safety, salt tolerance, bacteriocin production, and foreign plasmid restriction.

**Electronic supplementary material:**

The online version of this article (10.1186/s12864-018-4532-1) contains supplementary material, which is available to authorized users.

## Background

Jeotgal is the name used to collectively describe traditional Korean high-salt-fermented seafood products, which are made with seafood such as shrimps, oysters, and anchovies. It is often eaten as a side dish, used as a seasoning for kimchi and various soups, or as a dipping sauce with pork dishes. Jeotgal is made by adding up to 30% (w/w) sea salt to various types of seafood, and becomes palatable through autolysis and microbial enzyme activities during subsequent fermentation, attaining rich flavors and physical structures. Studies of the microbial structure and function in jeotgal have been performed to better understand the exact role of jeotgal-derived microorganisms [1–3]. However, the exact function of microorganisms in jeotgal production remains unclear, and starter cultures have not been implemented in the jeotgal industry.

To develop a more complete overview of the bacterial community in jeotgal, we evaluated the bacterial communities in two representative types of jeotgal using culture-based methods [3]. Coagulase-negative staphylococci (CNS) was identified as a major bacterial group, while *Staphylococcus equorum* was identified as the most dominant species. *S. equorum* has also frequently been detected in high-salt fermented meat products and cheeses in Europe [4–8]. It produces low molecular weight aroma compounds, such as esters, amino acids, aldehydes, and free fatty acids, through proteolysis and lipolysis in fermented foods. For this reason, *S. equorum* has been investigated as a component of starter cultures for fermentation of high-salt foods [9–13]. However, a small number of nosocomial infection cases caused by CNS species necessitates a safety assessment of potential industrial strains prior to their implementation in fermented food production [14].

*S. equorum* strain KS1039 was selected as a starter candidate from among many jeotgal-derived *S. equorum* strains after a series of safety assessments [9]. KS1039 was sensitive to 14 antibiotics, and was negative for hemolytic activity, biofilm formation, and biogenic amine production. Moreover, KS1039 exhibited efficient enzymatic activities, including protease and lipase activities, which are involved in the enhancement of sensory properties, and grew at a NaCl concentration of 25% (w/v) [9]. In our recent comparative genomic analysis of KS1039 with five other *S. equorum* strains, we identified the genomic basis of its safety and salt tolerance [1]. However, a comprehensive picture of the cellular components and metabolic pathways involved in the degradation of macromolecules and the development of sensory properties has not been proposed for *S. equorum*. Therefore, in this study, we examined the general metabolism of *S. equorum* based on information obtained from the published complete genome sequences of six *S. equorum* strains isolated from different niches. Additionally, the superiority of KS1039 as a starter culture for high-salt food fermentation was illuminated through comparative genomic analysis.

## Methods

### Bacterial strains and culture conditions

*S. equorum* strain KS1039, originally isolated from jeotgal, was used in this study. The genome of KS1039 was fully sequenced in our previous study [15], and these sequencing results were used in the current study to deduce the metabolic pathways present in *S. equorum*. To experimentally validate the sequencing results, four additional *S. equorum* strains, KM1031 from Myeolchi-jeotgal, C2014 from Saeu-jeotgal, Mu2 from French smear-ripened cheese [16], and UMC-CNS-924 from bovine mastitis [17], were used. *S. equorum* strains were cultured in tryptic soy broth/agar (TSB/TSA) at 30 °C for 24 h.

### Comparative genomics

For comparative genomic analysis of the genome of KS1039 (GenBank accession number CP013114.1), the complete genome sequences of *S. equorum* KM1031 (NZ_CP013980.1), *S. equorum* C2014 (NZ_CP013714.1), *S. equorum* subsp. *equorum* Mu2 (CAJL00000000.1), *S. equorum* UMC-CNS-924 (AVBD00000000.1), and *S. equorum* G8HB1 (LAKE00000000.1) were obtained from the NCBI Microbial Genomes database. The average nucleotide identities of the conserved genes among the genomes were identified using the Basic Local Alignment Search Tool (BLAST), and the data was used for comparative analysis [18]. Genome sequences of the *S. equorum* species involved in this study were uploaded to the Rapid Annotations using Subsystems Technology (RAST) server for SEED-based automated annotation, whole-genome sequence-based comparative analysis, and Kyoto Encyclopedia of Genes and Genomes (KEGG) metabolic pathway analysis [19]. Efficient Database framework for comparative Genome Analyses using BLAST score Ratios (EDGAR) was used for core genome, pan genome, and singleton analysis using *S. equorum* KS1039 as the reference genome [15]. Further comparative analyses were performed for specific regions and genes-of-interest using the BLASTN, BLASTX, and BLASTP tools.

### Physiological characterization

Strains were characterized biochemically using the commercially available ID 32 STAPH system according to the manufacturer’s instructions (bioMérieux).

### Determination of bacteriocin activity

The inhibitory activity of KS1039, along with that of the other *S. equorum* strains, was determined using the disk diffusion method on TSA agar at 30 °C for 24 h, using *Staphylococcus aureus* RN4220 as an indicator. An overnight culture of *S. aureus* RN4220 grown in TSB was used to inoculate fresh TSB medium to a final concentration of 1% (v/v), and then incubated at 30 °C to an optical density at 600 nm (OD_600_) of 1.0. The culture (200 μL) was then poured onto TSA plates. Sterile paper disks were placed on the surface of each plate. *S. equorum* strains were cultured to stationary phase and then diluted in TSB to an OD_600_ of 1.0. This dilution was centrifuged at 5000×*g* for 30 min at 4 °C, and the resulting supernatant was filtered using a 0.22-μm filter. A 15-μL aliquot of each of the supernatants was dropped onto separate disks on the TSA plates, and the antibacterial activity of each strain against *S. aureus* RN4220 was determined by halo formation.

### Plasmid electroporation

*S. equorum* was transformed by electroporation as described by Löfblom et al. [20]. Briefly, overnight cultures of *S. equorum* KS1039, as well as the other four strains, were inoculated into fresh TSB medium and incubated to an OD_600_ of 0.5. The cultures were chilled in an ice slurry for 10 min, with all subsequent steps performed at 4 °C on ice, and then harvested at 4000×*g* for 10 min. The pellets were resuspended in 1/10 volume of 0.5 M sucrose. The cells were then repeatedly centrifuged and resuspended, first in 1/10, then in 1/50, and finally in 1/100 the volume of ice-cold 0.5 M sucrose. Competent cells for electroporation (100 μL) were frozen at − 70 °C.

Two plasmids, pYJ335, containing an erythromycin resistance gene [21], and pCL55, containing a chloramphenicol resistance gene [22], were used to check the electroporation efficiency of KS1039 and the other four strains. The two plasmids were selected on growth media containing 10 μg/mL of erythromycin and 5 μg/mL of chloramphenicol, respectively. For electroporation, electrocompetent cells were thawed on ice for 5 min prior to the addition of 10 ng of plasmid DNA. The cells were incubated for 1 min on ice and then transferred to a chilled 2-mm electroporation cuvette (Bio-Rad) and pulsed at 25 kV/cm, 100 Ω, and 25 μF. A 900-μL volume of TSB was immediately added to the cells, which were then incubated at 30 °C for 2 h, before plating on TSA supplemented with the appropriate antibiotics. The plates were incubated at 37 °C for 24 h. Electroporation results were confirmed by three independently performed assays.

## Results and discussion

### Central carbohydrate metabolism of *S. equorum*

#### Glycolysis and tricarboxylic acid (TCA) cycle

**Fig. 1:**
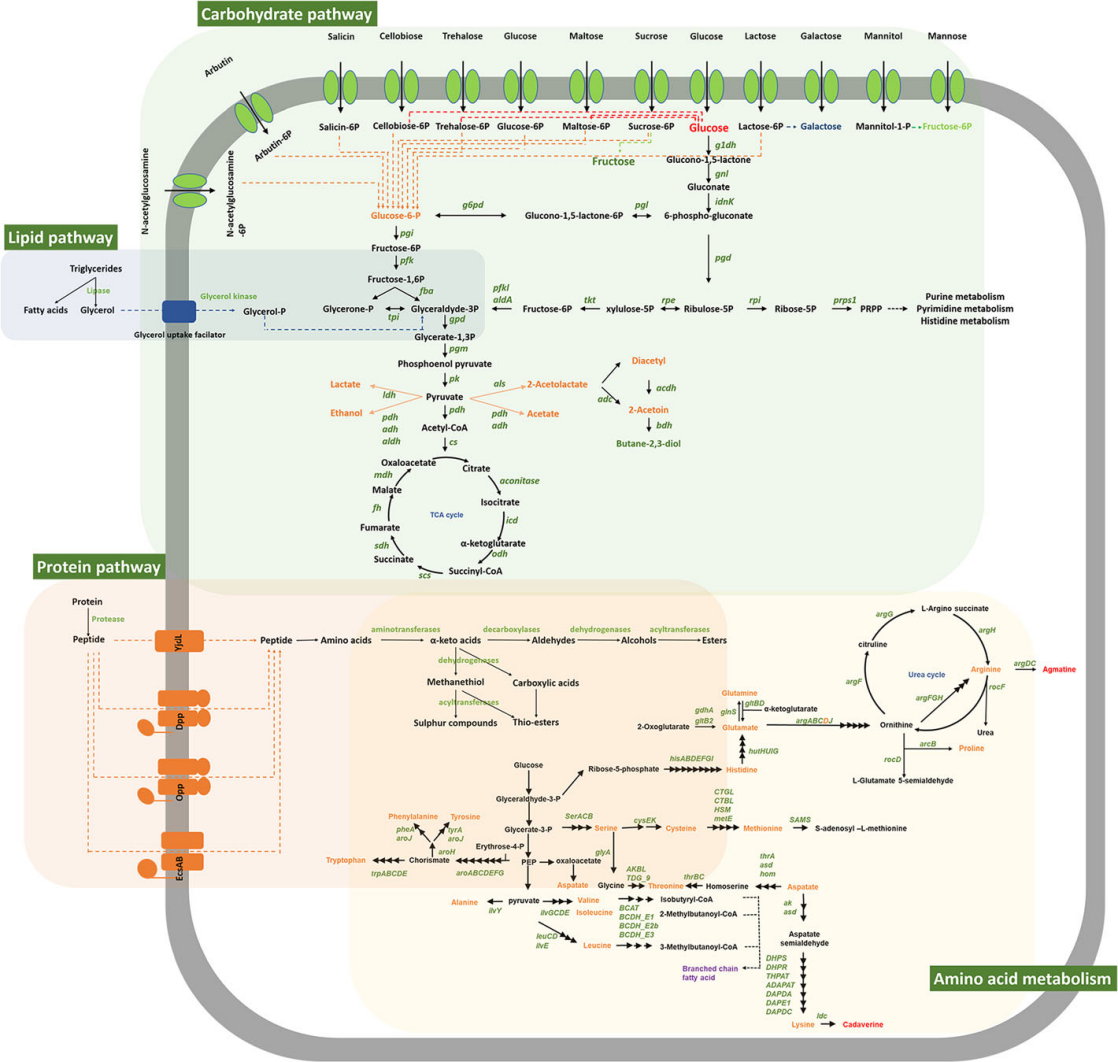
Predicted membrane transport systems and metabolic pathways for carbohydrates, proteins, and lipids in *Staphylococcus equorum.* The names of the enzyme-encoding genes are depicted in green. Metabolites involved in fermentation pathways are depicted in orange. Black arrows correspond to potential active enzymatic reactions catalyzed by the corresponding gene products encoded by the *S. equorum* genome

*S. equorum* KS1039 has the genetic potential to uptake glucose via either a phosphoenolpyruvate (PEP)-dependent phosphotransferase system (PTS) or a permease (Fig. 1, Additional file 1: Table S1). All genes encoding the enzymes involved in the glycolytic pathway (to pyruvate) were identified in the genome of *S. equorum* KS1039, as well as in the five other *S. equorum* strains (Fig. 1, Additional file 2: Table S2). All enzymes involved in the TCA cycle were also present in the six *S. equorum* strains (Additional file 2: Table S2).

#### Carbohydrate utilization

Different carbohydrate transport mechanisms were identified, including PEP-dependent sugar PTSs and permeases (Fig. 1). Genes for the utilization of cellobiose, maltose, mannose, mannitol, sucrose, and trehalose were also present, and these molecules could be taken up via a PTS; however, genes involved in raffinose, turanose, and sorbitol utilization were absent in *S. equorum* (Fig. 1 and Additional file 1: Table S1). Interestingly, the presence of a melibiose transporter gene was strain-specific. *S. equorum* had a fructose-specific PTS, but did not contain a permease gene, while a xylose-specific permease was detected along with a ribose-specific transporter (Additional file 1: Table S1).

*S. equorum* should be able to transport lactose via a lactose-specific PTS system. While *S. aureus*, *Staphylococcus epidermidis*, and *Staphylococcus hominis* can only utilize lactose via the tagatose-6-phosphate pathway [23], this pathway appeared to be missing in *S. equorum*. Instead, lactose-specific PTS and galactose-specific permease systems were detected in the genomes of *S. equorum*, along with two β-galactosidase genes (*S. equorum* KS1039 genome locus numbers SE1039_RS05770 and SE1039_RS12745).

To further investigate the carbohydrate utilization of the *S. equorum* strains, and to validate the genomic analysis results, we evaluated the biochemical characteristics of the five strains using an ID 32 STAPH system (Table 1). All five tested *S. equorum* strains utilized glucose, fructose, mannose, maltose, lactose, trehalose, mannitol, cellobiose, saccharose, and arabinose, which agreed with the results of the genetic analysis. Our results showed that three strains, KS1039, C2014, and Mu2, could not utilize raffinose, while the remaining two strains, KM1031 and UMC-CNS-924, could. KS1039 and Mu2 did not appear to contain the gene cluster required for melibiose/raffinose utilization (Additional file 3: Figure S1), or an α-galactosidase gene. Although C2014 did contain the α-galactosidase gene, a melibiose transporter was not identified in its genome sequence. *S. equorum* has been reported as a species that does not ferment raffinose [13]. However, we previously identifed a strain that could utilize raffinose [24], which is supported by the genetic information determined in this study.

**Table 1:** Phenotypic characteristics of *Staphylococcus equorum* isolates

Biochemical assay	KS1039	C2014	KM1031	Mu2	UMC-CNS-924
Fermentation					
D-glucose	+	+	+	+	+
D-fructose	+	+	+	+	+
D-mannose	+	+	+	+	+
D-maltose	+	+	+	+	+
D-lactose	+	+	+	+	+
D-trehalose	+	+	+	+	+
D-mannitol	+	+	+	+	+
D-raffinose	–	–	+	–	+
D-ribose	+	+	+	–	+
D-cellobiose	+	+	+	+	+
D-saccharose	+	+	+	+	+
D-turanose	–	–	–	–	–
L-arabinose	+	+	+	+	+
N-acetyl glucosamine	+	+	+	+	+
Reduction of nitrate	+	+	+	+	+
Production of acetoin	+	–	–	+	+
Urease	+	+	+	+	+
Ornithine decarboxylase	–	–	–	–	–
ß-Galactosidase	+	+	+	+	+
Arginine arylamidase	–	–	–	–	–
Alkaline phosphatase	–	–	–	–	–
Pyrrolidonyl arylamidase	–	–	–	–	–

#### Pentose phosphate pathway

Most genes involved in the pentose phosphate pathway were present in all six *S. equorum* strains (Additional file 4: Table S3). The strains contained gluconolactonase-encoding genes, and thus should be able to form 6-phosphgluconoate via glucono-1,5-lactone for glycolysis (Fig. 1). A ribose transport system was present in all six strains, while an ABC transporter gene was missing in Mu2 (Additional file 1: Table S1, Additional file 5: Figure S2), resulting in a lack of ribose utilization by Mu2 (Table 1). In the presence of ribose, *S. equorum* could produce important compounds via the pentose phosphate pathway by uptake of ribose. However, further studies are required to determine whether the lack of ribose transport is restricted to Mu2.

### Protein and amino acid metabolism of *S. equorum*

#### Amino acid biosynthesis

In silico prediction of the amino acid biosynthesis pathways in *S. equorum* was performed (Fig. 1, Additional file 6: Table S4). The genomes of the six strains appeared to contain loci coding for all of the enzymes required for the biosynthesis of all amino acids, with the exception of asparagine. Asparagine is synthesized from L-aspartate by asparagine synthase or via the aspartate-ammonia ligase reaction. However, the six *S. equorum* strains did not contain genes encoding any of the required enzymes, suggesting that *S. equorum* might be prototrophic for all amino acids except asparagine.

#### Proteolytic system

The six *S. equorum* strains contained a proteolytic system composed of secreted proteases, amino acid and peptide transport systems required for import, and a set of intracellular peptidases involved in the hydrolysis of peptides (Fig. 1). Multiple copies of serine protease and CAAX amino terminal protease genes were detected (Additional file 7: Table S5). The serine protease was similar to the well-characterized secreted protease SspA from *S. aureus*, which is proteolytically cleaved to produce a mature and functional enzyme [25]. CAAX amino terminal proteases demonstrate protease activity when anchored to the membrane [26]. Therefore, we assumed that these proteases play a role in protein degradation.

#### Amino acid and peptide transport systems

There is limited experimental data on *S. equorum* amino acid and peptide transport systems. Although no amino acid or di-tripeptide transport systems have been characterized in detail in *S. equorum*, this species is frequently detected in protein-rich fermented foods [13, 16], and has been linked to aroma production from amino acids [11]. Therefore, characterization of any oligopeptide ABC transport systems in *S. equorum* would be useful to help explain the role of this species in fermented foods.

The genomes of the six *S. equorum* strains contained a proton-dependent di-tripeptide transporter system, YjdL. In addition, three ABC transport systems, Opp, EcsAB, and Dpp, were detected (Fig. 1, Additional file 8: Table S6). The Opp transporter is composed of an oligopeptide ABC transporter (OppA), two membrane proteins that form the permease (OppB and OppC), and two ATP-binding proteins (OppD and OppF) that provide energy for the system. The *oppABCDF* genes were organized in an operon. The Ecs transporter is also composed of an ABC transporter (EcsA), permease proteins (EcsB), and a protein of unknown function (EcsC) that has been suggested to be involved in peptide transport [27]. In the six strains, *ecsA* and *ecsB* were detected in the absence of *ecsC*. In addition, the Dpp transporter operon in these strains appeared to be incomplete, with only *dppC* and *dppD* being detected. Despite these findings, the main systems for peptide and amino acid transport were identified in the genomes of the *S. equorum* strains, although some of the genes appeared to have been subjected to breakage, rearrangement, and duplication processes. Consequently, we hypothesize that the Opp transport system is likely to play the greatest role in peptide transport into the cell.

### Characteristics of *S. equorum* involved in food fermentations

#### Color development

*S. equorum* plays a role in color development of fermented meat by the reduction of nitrates to nitrite, and then to nitrous oxide [28]. Related gene clusters, nitrate reductase (*nar*), nitrous oxide reductase (*nor*), and nitrite reductase (*nir*), were detected in the *S. equorum* KS1039 genome, as well as in other strains (Additional file 9: Figure S3). The nitrate reductase activity of *S. equorum* KS1039 has previously been reported in laboratory media supplemented with nitrate [9].

#### Flavor development

Butane-2,3-diol are produced from low molecular-weight volatile compounds such as diacetyl and acetoin by microorganisms. Lactic acid bacteria produce both diacetyl and acetoin, which are recognized as buttery aroma compounds. According to the genomic analysis performed in the current study, most *S. equorum* strains possess genes involved in the production of these compounds. They appear to be generated via NAD^+^ regeneration from pyruvate using three genes: *als* (α-acetolactate synthase), *adc* (α-acetolactate decarboxylase), and *bdh* (2,3-butanediol dehydrogenase) (Fig. 1, Additional file 2: Table S2). *adc* was not identified in C2014. Pyruvate may be converted into acetate, lactate, and ethanol, all of which contribute to the sensory properties. All of the *S. equorum* strains contained the genes responsible for the production of these three products.

In addition, the catabolism of amino acids plays an important role in providing precursors for the biosynthesis of amino acids, nucleotides, and vitamins, generating energy in nutrient-limited environments, increasing intracellular pH, and production of ester and sulfur compounds. These compounds contribute to the aroma of fermented foods. Catabolism of amino acids is commonly initiated by a transamination step, requiring the presence of α-ketoglutarate as the amino group acceptor, to produce α-keto acids. All of the *S. equorum* genomes contained several aminotransferase genes, including two aspartate aminotransferases (SE1039_RS08735 and SE1039_RS08745), one aromatic aminotransferase (SE1039_RS02785), and one branched chain amino acid aminotransferase (SE1039_RS01825). Two glutamate dehydrogenase genes (SE1039_RS01790 and SE1039_RS03600) are required to produce α-ketoglutarate from amino acid transamination (Fig. 1). Experimental data showed that *S. equorum* produced methyl-branched ketones, compounds involved in aroma, from leucine, isoleucine, and valine [11]. These results supported the data generated from the genetic analysis of *S. equorum* (Fig. 1).

#### Absence of biogenic amine production

Recently, several starter candidates have been selected to reduce the safety hazards involved with fermentation [29–34]. Biogenic amines produced by naturally occurring microorganisms are significant safety hazards in protein-rich fermented foods. The major biogenic amines found in foods are putrescine, cadaverine, histamine, tyramine, tryptamine, 2-phenyl-ethylamine, spermine, agmatine, and spermidine. The formation of biogenic amines in fermented foods has been attributed to the decarboxylase activity of bacteria involved in fermentation [35]. Among the biogenic amines, histamine and tyramine are hazardous to human health because of their vasoactive and psychoactive properties [36]. Although none of the enzymes involved in decarboxylation of histidine and tyramine were identified in the genomes of the six *S. equorum* strains, the lysine decarboxylase gene (SE1039_RS01180), which is involved in cadaverine production, was identified. However, our previous study showed that KS1039 does not produce cadaverine, histamine, putrescine, or tyramine [9]. Other coagulase-negative staphylococci isolated from doenjang, including *S. saprophyticus, S. succinus*, and *S. xylosus*, produced non-significant amounts of cadaverine and putrescine, but did not produce histamine and tyramine [36]. Therefore, the lack of genes related to histamine and tyramine production in the genome of *S. equorum* supports its potential implementation in food fermentation processes.

### Useful characteristics of KS1039 as a starter culture for food fermentations

#### Bacteriocin production

**Fig. 2:**
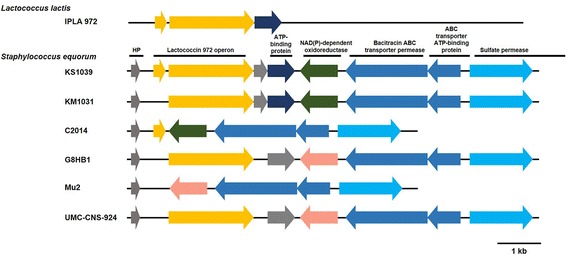
Genetic organization of the lactococcin 972 cluster from *Staphylococcus equorum* isolates

Production of bacteriocins, such as epidermin, epicidin 280, hyicin 3682, and nukacin L217, has been reported in *S. epidermidis* Tu 3298, *S. epidermidis* BN280, *Staphylococcus hycius* 3682, and *Staphylococcus chromogenes* L217, respectively [37–40]. KS1039 contained the entire lactococcin 972 operon, including the pre-bacteriocin (SE1039_RS11380) and immunity protein (SE1039_RS11385) genes (Fig. 2). The lactococcin 972 operon, a bacteriocin gene cluster, was first identified in *Lactococcus lactis*, where the corresponding proteins had a bactericidal effect on sensitive lactococci [41]. The lactococcin 972 operon encodes a pre-bacteriocin protein that forms a homodimer that is lethal to target strains, as well as an immunity protein that protects the producing cell from the action of the bacteriocin. Unlike KS1039, the operon was not present in Mu2, while the four other strains, KM1031, C2014, G8HB1, and UMC-CNS-924, possessed one or other of the two genes (Fig. 2). Expression of the lactococcin 972 operon in KS1039 was confirmed experimentally by examining the antibacterial activity of *S. equorum* strains against *S. aureus* (Additional file 10: Figure S4).

Consumers frequently demand additional health properties from fermented foods, such as prevention of food poisoning. Therefore, a bacterial strain with bacteriocin-producing abilities, such as KS1039, has a unique advantage as a starter culture for fermented food production, as it would function as a natural preservative and prevent the growth of bacterial pathogens.

#### Clustered regularly interspaced short palindromic repeat (CRISPR)/Cas systems

**Fig. 3:**
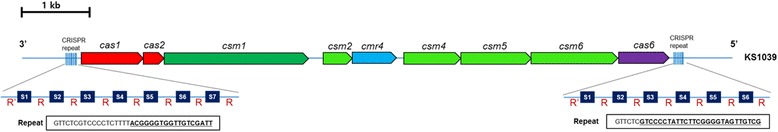
CRISPR/Cas system of *Staphylococcus equorum* KS1039

CRISPR/Cas systems have the ability to acquire short pieces of DNA (spacers) that provide immunity against subsequent exposure to phages and plasmids carrying matching sequences [42–44]. The detailed mechanism by which CRISPR/Cas systems provide resistance to foreign DNA is currently the subject of much investigation. Several CRISPR/Cas systems have been identified in *Streptococcus thermophilus*, and are assumed to aid in the survival of the bacterium in milk [45]. The CRISPR/Cas systems of *S. thermophilus* decreased the uptake and dissemination of undesirable plasmid-encoded genetic elements in *Escherichia coli* [46]. CRISPR/Cas sequences were detected in the genome of KS1039 (SE1039_RS13110–SE1039_RS13150), but were not present in the other five published *S. equorum* genomes (C2014, KM1031, Mu2, UMC-CNS-924, and G8HB1). The KS1039 CRISPR/Cas region spans approximately 8.5 kb, and consists of nine *cas* genes: *cas1*, *cas2*, *csm1*, *csm2*, *cmr4*, *csm4*, *csm5*, *csm6*, and *cas6* (Fig. 3). Generally, CRISPR consist of short conserved repeat sequences interspaced by unique DNA sequences of similar size, called spacers. Two different repeat sequences with unique DNA sequences were detected in the flanking regions of *cas* genes in KS1039. It was reported that the unique DNA sequences originate from phage or plasmid DNA, and when foreign genes containing unique DNA sequences are inserted into bacteria, Cas proteins interfere with the invasive DNA through digestion during recombination between the invasive and host DNA [43, 44, 46, 47]. Therefore, we hypothesize that the CRISPR/Cas system could prevent the acquisition of foreign virulence genes by KS1039, which does not possess any known virulence genes [1].

To prove our hypothesis, we checked the transformation efficiency of KS1039 using plasmids pYJ335 and pCL55 (Table 2). Interestingly, no KS1039 transformants were obtained for either plasmid, whereas pYJ335 was transformed into the other *S. equorum* strains at frequencies of 5.9 × 10^− 4^ to 5.2 × 10^− 8^ colony-forming units (cfu)/ml. In particular, multidrug-resistant strain KM1031 showed high transformation efficiency. As plasmid pCL55 carries a chloramphenicol resistance marker, transformation experiments using this plasmid could not be carried out in strain KM1031, which shows resistance to chloramphenicol. However, pCL55 could be transformed into strains C2014, Mu2, and UMC-CNS-924 at frequencies of 5.8 × 10^− 8^, 2.4 × 10^− 6^, and 4.5 × 10^− 8^ cfu/ml, respectively. These results supported the hypothesis that CRISPR/Cas sequences in KS1039 prevent plasmid acquisition, again suggesting that *S. equorum* KS1039 is a good starter candidate for fermented foods.

**Table 2:** In vitro transformation efficiency of pYJ335 and pCL55 into *Staphylococcus equorum* strains

Strain	Transformation rate (T/C; cfu/ml)	
	pYJ335	pCL55
*S. equorum* KS1039	–	–
*S. equorum* C2014	4.0 × 10^−7^	5.8 × 10^−8^
*S. equorum* KM1031	5.9 × 10^−4^	–
*S. equorum* Mu2	1.4 × 10^−8^	2.4 × 10^−6^
*S. equorum* UMC-CNS-924	5.2 × 10^−8^	4.5 × 10^−8^

Cell counts represent the mean values of three independent replicates

T, transformed cell counts per 1 ml; C, competent cell count per 1 ml

Transformants were confirmed by plasmid identification

## Conclusions

Analyses of the genome sequences of *S. equorum* KS1039 and five other *S. equorum* strains accurately identified the presence of metabolic pathways for carbohydrates and proteins. Thus, these analyses could shed light on how certain strains contribute to food fermentation, and may be useful in selecting optimal starter candidates based on appropriate sensory properties. As a starter candidate, *S. equorum* KS1039 possessed useful gene clusters, including those for bacteriocin production and a CRISPR/Cas system, which were not present in the other strains. Experimental results confirmed that *S. equorum* KS1039 exhibited bacteriocin activity, and that the CRISPR/Cas system prevented the uptake of foreign plasmid DNA. Therefore, *S. equorum* KS1039 displays the desirable properties of a starter culture for the fermentation of high-salt foods with regards to safety, salt tolerance, bacteriocin production, and foreign plasmid restriction.

## Additional files

Additional file 1: **Table S1.** List of genes involved in sugar transport systems. (DOCX 25 kb)

Additional file 2: **Table S2.** List of genes involved in carbohydrate metabolism. (DOCX 22 kb)

Additional file 3: **Figure S1.** Genetic organization of the genes involved in melibiose/raffinose utilization in *S. equorum*. (DOCX 48 kb)

Additional file 4: **Table S3.** List of genes involved in the pentose phosphate pathway. (DOCX 20 kb)

Additional file 5: **Figure S2.** Genetic organization of the ribose operon in *S. equorum*. (DOCX 70 kb)

Additional file 6: **Table S4.** List of genes involved in amino acid biosynthesis. (DOCX 33 kb)

Additional file 7: **Table S5.** List of genes coding for proteases and peptidases. (DOCX 20 kb)

Additional file 8: **Table S6.** List of the genes involved in protein and peptide transport systems. (DOCX 19 kb)

Additional file 9: **Figure S3.** Genetic organization of nitrogen metabolism in *S. equorum*. (DOCX 51 kb)

Additional file 10: **Figure S4.** Growth inhibition of *S. aureus* by *S. equorum* strains. *S. aureus* RN4220 was used as an indicator strain. (DOCX 336 kb)

## Abbreviations

BLAST: Basic Local Alignment Search Tool
CNS: Coagulase-negative staphylococci
CRISPR: Clustered regularly-interspaced short palindromic repeat
EDGAR: Efficient Database framework for comparative Genome Analyses using BLAST score Ratios
KEGG: Kyoto Encyclopedia of Genes and Genomes
PEP: Phosphoenolpyruvate
PTS: Phosphotransferase system
RAST: Rapid Annotations using Subsystems Technology
TCA: Tricarboxylic acid
TSA: Tryptic soy agar
TSB: Tryptic soy broth
