# Supplementary material for: Genomic insights into Staphylococcus equorum KS1039 as a potential starter culture for the fermentation of high-salt foods
Source: BMC Genomics. 2018 Feb 13;19:136. doi: 10.1186/s12864-018-4532-1 (PMC5810056; doi:10.1186/s12864-018-4532-1)
Supplement: Supplementary file 1 — Table S1. List of genes involved in sugar transport systems. (DOCX 25 kb) [file 12864_2018_4532_MOESM1_ESM.docx]

Table S1. List of genes involved in sugar transport systems.

| **Target** | **Transport system** | **KS1039**  **locus tags** | **C2014** | **KM1031** | **G8HB1** | **Mu2** | **UMC-CNS-924** |
| --- | --- | --- | --- | --- | --- | --- | --- |
| Glucose | Glucose transporter | SE1039_RS09780 | AVJ22_RS09750 | AWC34_RS09355 | UF72_RS08805 | SEQMU2_RS01640 | SEQU_RS14045 |
|  | PTS glucose transporter subunit IIBC | SE1039_RS07715 | AVJ22_RS07570 | AWC34_RS07300 | UF72_RS06065 |  | SEQU_RS18795 |
|  | PTS glucose transporter subunit IIB | SE1039_RS11800 | AVJ22_RS11685 | AWC34_RS11380 | UF72_RS10840 | SEQMU2_RS03645 | SEQU_RS17315 |
|  | PTS glucose transporter subunit IIABC | SE1039_RS11220 | AVJ22_RS11185 | AWC34_RS10800 | UF72_RS10255 | SEQMU2_RS03095 | SEQU_RS15490 |
|  | PTS glucose transporter subunit IIA | SE1039_RS06325 | AVJ22_RS06135 | AWC34_RS05890 | UF72_RS04670 | SEQMU2_RS11435 | SEQU_RS19695 |
| Fructose | PTS fructose transporter subunit IIC | SE1039_RS02675 | AVJ22_RS02510 | AWC34_RS02725 | UF72_RS01185 | SEQMU2_RS07465 | SEQU_RS21485 |
| Sucrose | PTS sucrose transporter subunit IIBC | SE1039_RS10465 | AVJ22_RS10450 | AWC34_RS10040 | UF72_RS09495 | SEQMU2_RS02325 | SEQU_RS14730 |
|  | PTS system, sucrose-specific IIB component | SE1039_RS10085 | AVJ22_RS10065 | AWC34_RS09660 | UF72_RS09115 | SEQMU2_RS01945 | SEQU_RS14350 |
| Mannose | PTS mannose transporter subunit IIABC | SE1039_RS11645 | AVJ22_RS11575 | AWC34_RS11240 | UF72_RS10695 | SEQMU2_RS03500 | SEQU_RS17170 |
|  | PTS mannose transporter subunit IIA | SE1039_RS00730 | AVJ22_RS00640 | AWC34_RS00650 |  | SEQMU2_RS06255 | SEQU_RS23015 |
|  | PTS mannose transporter subunit IIA | SE1039_RS11745 | AVJ22_RS11640 | AWC34_RS11340 | UF72_RS10795 | SEQMU2_RS03600 | SEQU_RS17270 |
|  | PTS mannose transporter subunit IIA | SE1039_RS02440 | AVJ22_RS11580 | AWC34_RS11245 | UF72_RS00945 | SEQMU2_RS07225 | SEQU_RS21240 |
| Mannitol | PTS mannitol transporter subunit IIB | SE1039_RS09385 | AVJ22_RS09310 | AWC34_RS08960 | UF72_RS12030 | SEQMU2_RS01260 | SEQU_RS24500 |
|  | PTS mannitol transporter subunit IIA | SE1039_RS09395 | AVJ22_RS09320 | AWC34_RS08970 | UF72_RS12040 | SEQMU2_RS01270 | SEQU_RS24510 |
| Ribose | Ribose pyranase | SE1039_RS05925 | AVJ22_RS05770 | AWC34_RS05535 | UF72_RS04325 | SEQMU2_RS11015 | SEQU_RS17015 |
|  | ABC transporter | SE1039_RS05930 | AVJ22_RS05775 | AWC34_RS05540 | UF72_RS04330 |  | SEQU_RS17020 |
|  | Ribose ABC transporter permease | SE1039_RS05935 | AVJ22_RS05780 | AWC34_RS05545 | UF72_RS04335 | SEQMU2_RS11025 | SEQU_RS17025 |
|  | D-ribose transporter subunit RbsB | SE1039_RS05940 | AVJ22_RS05785 | AWC34_RS05550 | UF72_RS04340 | SEQMU2_RS11030 | SEQU_RS17030 |
|  | Ribose transporter RbsU | SE1039_RS12485 | AVJ22_RS12405 | AWC34_RS12030 | UF72_RS08265 | SEQMU2_RS04280 | SEQU_RS17930 |
|  | Ribose pyranase | SE1039_RS12490 | AVJ22_RS12410 | AWC34_RS12035 | UF72_RS08260 | SEQMU2_RS04285 | SEQU_RS17935 |
| Lactose | PTS lactose transporter subunit IIB | SE1039_RS11730 | AVJ22_RS09315 | AWC34_RS08965 | UF72_RS10780 | SEQMU2_RS03585 | SEQU_RS17255 |
|  | PTS sugar transporter subunit IIA | SE1039_RS12560 | AVJ22_RS11635 | AWC34_RS11335 | UF72_RS08180 | SEQMU2_RS04375 | SEQU_RS18035 |
|  | PTS galactitol transporter subunit IIC | SE1039_RS12570 | AVJ22_RS12520 | AWC34_RS12125 | UF72_RS08170 | SEQMU2_RS04385 | SEQU_RS18045 |
|  | PTS galactitol transporter subunit IIB | SE1039_RS12565 | AVJ22_RS12515 | AWC34_RS12120 | UF72_RS08175 | SEQMU2_RS04380 | SEQU_RS18040 |
|  | PTS system, Lactose/Cellobiose specific IIB subunit | SE1039_RS00720 | AVJ22_RS00630 | AWC34_RS00640 | UF72_RS06805 | SEQMU2_RS06245 | SEQU_RS23005 |
| Cellobiose | PTS cellobiose transporter subunit IIC | SE1039_RS11760 |  | AWC34_RS11355 | UF72_RS10810 | SEQMU2_RS03615 | SEQU_RS17285 |
|  | PTS cellobiose transporter subunit IIB | SE1039_RS11750 | AVJ22_RS05680 | AWC34_RS05455 | UF72_RS10800 | SEQMU2_RS03605 | SEQU_RS17275 |
|  | PTS system, cellobiose-specific IIC component | SE1039_RS00725 | AVJ22_RS00635 | AWC34_RS00645 | UF72_RS06800 | SEQMU2_RS06250 | SEQU_RS23010 |
| Galactose | Galactose:cation symporter | SE1039_RS00045 | AVJ22_RS00045 | AWC34_RS00045 | UF72_RS07395 | SEQMU2_RS05640 | SEQU_RS20745 |
| Maltose | PTS alpha-glucoside transporter subunit IIBC | SE1039_RS02150 | AVJ22_RS01980 | AWC34_RS02190 | UF72_RS00655 | SEQMU2_RS06935 | SEQU_RS22685 |
|  | PTS alpha-glucoside transporter subunit IIBC | SE1039_RS10140 | AVJ22_RS10120 | AWC34_RS09715 | UF72_RS09170 | SEQMU2_RS06935 | SEQU_RS14405 |
| Melibiose | Na+/melibiose symporter |  |  | AWC34_RS12365 | UF72_RS06885 |  | SEQU_RS22925 |
| Arabinose | Arabinose-proton symporter | SE1039_RS12455 | AVJ22_RS12375 | AWC34_RS12000 | UF72_RS08295 | SEQMU2_RS02235 | SEQU_RS17900 |
| Trehalose | PTS system, trehalose-specific IIB component | SE1039_RS01110 | AVJ22_RS00390 | AWC34_RS00430 | UF72_RS06450 | SEQMU2_RS06695 | SEQU_RS23365 |
| Xylose | D-xylose proton-symporter XylE | SE1039_RS12355 | AVJ22_RS12275 | AWC34_RS11955 | UF72_RS08340 | SEQMU2_RS04205 | SEQU_RS17880 |
| Glycerol | Glycerol transporter | SE1039_RS05600 | AVJ22_RS05130 | AWC34_RS05240 | UF72_RS04030 | SEQMU2_RS10450 | SEQU_RS16720 |
| Sugar | PTS system sugar-specific permease component | SE1039_RS11725 | AVJ22_RS11620 | AWC34_RS11320 | UF72_RS10775 | SEQMU2_RS03580 | SEQU_RS17250 |
|  | Sugar symporter | SE1039_RS12750 | AVJ22_RS12185 | AWC34_RS11895 | UF72_RS07930 | SEQMU2_RS04625 | SEQU_RS18280 |
|  | Predicted galactitol operon regulator | SE1039_RS11735 | AVJ22_RS11630 | AWC34_RS11330 | UF72_RS10785 | SEQMU2_RS03590 | SEQU_RS17260 |
|  | D-glucuronide-specific TRAP transporter | SE1039_RS01745 | AVJ22_RS01600 | AWC34_RS01680 | UF72_RS00365 | SEQMU2_RS00255 | SEQU_RS23485 |
|  | Sugar transporter | SE1039_RS10375 | AVJ22_RS10355 | AWC34_RS09950 | UF72_RS09405 | SEQMU2_RS02235 | SEQU_RS14640 |
|  | Sugar transporter | SE1039_RS10940 | AVJ22_RS04990 | AWC34_RS05085 | UF72_RS09975 | SEQMU2_RS02820 | SEQU_RS15210 |
