# Supplementary material for: Genomic insights into Staphylococcus equorum KS1039 as a potential starter culture for the fermentation of high-salt foods
Source: BMC Genomics. 2018 Feb 13;19:136. doi: 10.1186/s12864-018-4532-1 (PMC5810056; doi:10.1186/s12864-018-4532-1)
Supplement: Supplementary file 2 — Table S2. List of genes involved in carbohydrate metabolism. (DOCX 22 kb) [file 12864_2018_4532_MOESM2_ESM.docx]

Table S2. List of genes involved in carbohydrate metabolism.

| **Enzyme** | **Gene name** | **KS1039**  **locus tags** | **C2014** | **KM1031** | **G8HB1** | **Mu2** | **UMC-CNS-924** |
| --- | --- | --- | --- | --- | --- | --- | --- |
| Phosphoglucose isomerase | *pgi* | SE1039_RS03635 | AVJ22_RS03460 | AWC34_RS03670 | UF72_RS02165 | SEQMU2_RS08860 | SEQU_RS21940 |
| 6-Phosphofructokinase | *pfk* | SE1039_RS07560 | AVJ22_RS07415 | AWC34_RS07145 | UF72_RS05910 | SEQMU2_RS12675 | SEQU_RS18950 |
| Fructose-1,6-bisphosphate aldolase | *fba* | SE1039_RS11960 | AVJ22_RS09145 | AWC34_RS11545 | UF72_RS11000 | SEQMU2_RS03805 | SEQU_RS17470 |
| Triosephosphate isomerase | *tpi* | SE1039_RS03065 | AVJ22_RS02910 | AWC34_RS03115 | UF72_RS01575 | SEQMU2_RS08170 | SEQU_RS25175 |
| Glyceraldehyde-3-phosphate dehydrogenase | *gpd* | SE1039_RS07510 | AVJ22_RS03590 | AWC34_RS03795 | UF72_RS05860 | SEQMU2_RS12625 | SEQU_RS19000 |
| Phosphoglycerate mutase | *pgm* | SE1039_RS00790 | AVJ22_RS00710 | AWC34_RS00710 | UF72_RS06735 | SEQMU2_RS06315 | SEQU_RS23075 |
| Pyruvate kinase | *pk* | SE1039_RS07555 | AVJ22_RS07410 | AWC34_RS07140 | UF72_RS05905 | SEQMU2_RS12670 | SEQU_RS18955 |
| Pyruvate dehydrogenase (E1 subunit alpha) | *pdh* | SE1039_RS04265 | AVJ22_RS04090 | AWC34_RS04260 | UF72_RS02755 | SEQMU2_RS09475 | SEQU_RS15745 |
| Citrate synthase | *cs* | SE1039_RS07545 | AVJ22_RS07400 | AWC34_RS07130 | UF72_RS05895 | SEQMU2_RS12660 | SEQU_RS18965 |
| Aconitase (aconitate hydratase) | *aconitase* | SE1039_RS05855 | AVJ22_RS05695 | AWC34_RS05470 | UF72_RS04260 | SEQMU2_RS10950 | SEQU_RS16950 |
| Isocitrate dehydrogenase | *icd* | SE1039_RS07540 | AVJ22_RS07395 | AWC34_RS07125 | UF72_RS05890 | SEQMU2_RS12655 | SEQU_RS18970 |
| 2-Oxoglutarate dehydrogenase | *odh* | SE1039_RS06285 | AVJ22_RS06095 | AWC34_RS05850 | UF72_RS04630 | SEQMU2_RS11395 | SEQU_RS19655 |
| Succinate dehydrogenase | *sdh* | SE1039_RS04485 | AVJ22_RS04310 | AWC34_RS04480 | UF72_RS02975 | SEQMU2_RS09695 | SEQU_RS15965 |
|  |  | SE1039_RS04490 | AVJ22_RS04315 | AWC34_RS04485 | UF72_RS02980 | SEQMU2_RS09700 | SEQU_RS15970 |
|  |  | SE1039_RS04495 | AVJ22_RS04320 | AWC34_RS04490 | UF72_RS02985 | SEQMU2_RS09705 | SEQU_RS15975 |
| Succinyl-CoA synthetase | *scs* | SE1039_RS05300 | AVJ22_RS04850 | AWC34_RS04945 | UF72_RS03735 | SEQMU2_RS10150 | SEQU_RS16425 |
| Fumarate hydratase | *fh* | SE1039_RS08210 | AVJ22_RS08080 | AWC34_RS07795 | UF72_RS13390 | SEQMU2_RS13340 | SEQU_RS26245 |
| Malate dehydrogenase | *mdh* | SE1039_RS12920 | AVJ22_RS12880 | AWC34_RS12505 | UF72_RS07790 | SEQMU2_RS04795 | SEQU_RS21040 |
| Aldehyde dehydrogenase | *aldh* | SE1039_RS08675 | AVJ22_RS08550 | AWC34_RS08255 | UF72_RS12980 | SEQMU2_RS00550 | SEQU_RS24860 |
| Alcohol dehydrogenase | *adh* | SE1039_RS09565 | AVJ22_RS09490 | AWC34_RS09140 | UF72_RS08590 | SEQMU2_RS01425 | SEQU_RS13790 |
| α-Acetolactate synthase | *als* | SE1039_RS08880 | AVJ22_RS08795 | AWC34_RS08455 | UF72_RS13185 | SEQMU2_RS00770 | SEQU_RS26020 |
| α-Acetolactate decarboxylase | *adc* | SE1039_RS10995 |  | AWC34_RS10570 | UF72_RS10030 | SEQMU2_RS02875 | SEQU_RS15265 |
| Acetoin dehydrogenase | *acdh* | SE1039_RS07750 | AVJ22_RS07605 | AWC34_RS07335 | UF72_RS06100 | SEQMU2_RS12875 | SEQU_RS18760 |
| Butanediol dehydrogenase | *bdh* | SE1039_RS10420 | AVJ22_RS00590 | AWC34_RS09995 | UF72_RS09450 | SEQMU2_RS02280 | SEQU_RS14685 |
| Lactate dehydrogenase | *ldh* | SE1039_RS07890 | AVJ22_RS07755 | AWC34_RS07475 | UF72_RS06255 | SEQMU2_RS13015 | SEQU_RS18620 |
