# Supplementary material for: Genomic insights into Staphylococcus equorum KS1039 as a potential starter culture for the fermentation of high-salt foods
Source: BMC Genomics. 2018 Feb 13;19:136. doi: 10.1186/s12864-018-4532-1 (PMC5810056; doi:10.1186/s12864-018-4532-1)

**Fig S1.** Genetic organization of the genes involved in melibiose/raffinose utilization in *S. equorum*.


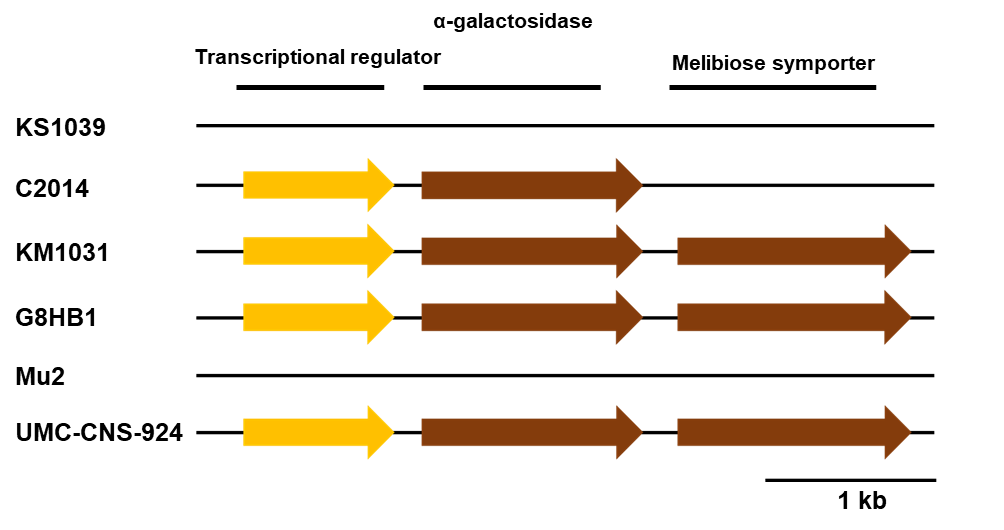

Supplement: Supplementary file 3 — Figure S1. Genetic organization of the genes involved in melibiose/raffinose utilization in S. equorum. (DOCX 48 kb) [file 12864_2018_4532_MOESM3_ESM.docx]
