# Supplementary material for: Genomic insights into Staphylococcus equorum KS1039 as a potential starter culture for the fermentation of high-salt foods
Source: BMC Genomics. 2018 Feb 13;19:136. doi: 10.1186/s12864-018-4532-1 (PMC5810056; doi:10.1186/s12864-018-4532-1)
Supplement: Supplementary file 4 — Table S3. List of genes involved in the pentose phosphate pathway. (DOCX 20 kb) [file 12864_2018_4532_MOESM4_ESM.docx]

Table S3. List of genes involved in the pentose phosphate pathway.

| **Enzyme** | **Gene name** | **KS1039 locus tags** | **C2014** | **KM1031** | **G8HB1** | **Mu2** | **UMC-CNS-924** |
| --- | --- | --- | --- | --- | --- | --- | --- |
| Glucose-1-dehydrogenase | *g1dh* | SE1039_RS09785 | AVJ22_RS09755 | AWC34_RS09360 | UF72_RS08810 | SEQMU2_RS01645 | SEQU_RS14050 |
| Gluconolactonase | *gnl* | SE1039_RS08680 | AVJ22_RS04170 | AWC34_RS04335 | UF72_RS12985 | SEQMU2_RS00555 | SEQU_RS24865 |
|  |  | SE1039_RS11255 | AVJ22_RS11220 | AWC34_RS10845 | UF72_RS10300 |  | SEQU_RS23845 |
|  |  | SE1039_RS00085 | AVJ22_RS00085 | AWC34_RS00085 | UF72_RS07355 | SEQMU2_RS05680 | SEQU_RS20705 |
| Gluconokinase | *idnK* | SE1039_RS10960 | AVJ22_RS10930 | AWC34_RS10535 | UF72_RS09995 | SEQMU2_RS02840 | SEQU_RS15230 |
|  |  | SE1039_RS06250 | AVJ22_RS02000 | AWC34_RS05820 | UF72_RS04600 | SEQMU2_RS11300 | SEQU_RS19625 |
|  |  | SE1039_RS12370 | AVJ22_RS12290 | AWC34_RS11970 | UF72_RS00350 | SEQMU2_RS00240 | SEQU_RS23500 |
| 6-Phosphogluconate dehydrogenase | *pgd* | SE1039_RS06685 | AVJ22_RS06515 | AWC34_RS06255 | UF72_RS05030 | SEQMU2_RS11795 | SEQU_RS20055 |
| Ribose 5-phosphate isomerase A | *rpi* | SE1039_RS10210 | AVJ22_RS10190 | AWC34_RS09785 | UF72_RS09240 | SEQMU2_RS02070 | SEQU_RS14475 |
| Ribose-phosphate pyrophosphokinase | *prps1* | SE1039_RS01275 | AVJ22_RS01140 | AWC34_RS01165 | UF72_RS12210 | SEQMU2_RS13685 | SEQU_RS25535 |
| Ribulose-phosphate 3-epimerase | *rpe* | SE1039_RS05150 | AVJ22_RS04690 | AWC34_RS04795 | UF72_RS03585 | SEQMU2_RS10000 | SEQU_RS16275 |
| Transketolase | *tkt* | SE1039_RS05790 | AVJ22_RS10780 | AWC34_RS10375 | UF72_RS04220 | SEQMU2_RS10640 | SEQU_RS16910 |
| 6-Phosphofructokinase | *pfkl* | SE1039_RS07560 | AVJ22_RS07415 | AWC34_RS07145 | UF72_RS05910 | SEQMU2_RS12675 | SEQU_RS18950 |
| Fructose-bisphosphate aldolase | *aldA* | SE1039_RS09220 | AVJ22_RS09145 | AWC34_RS08795 | UF72_RS11865 | SEQMU2_RS01095 | SEQU_RS24335 |
|  |  | SE1039_RS11960 | AVJ22_RS11840 | AWC34_RS11545 | UF72_RS11000 | SEQMU2_RS03805 | SEQU_RS17470 |
| 6-Phosphogluconolactonase | *pgl* | SE1039_RS08680 | AVJ22_RS08555 | AWC34_RS08260 | UF72_RS12985 | SEQMU2_RS00555 | SEQU_RS24865 |
| Glucose-6-phosphate 1-dehydrogenas | *g6pd* | SE1039_RS06665 | AVJ22_RS06490 | AWC34_RS06235 | UF72_RS05010 | SEQMU2_RS11775 | SEQU_RS20035 |
