# Supplementary figures and images for: Genomic insights into Staphylococcus equorum KS1039 as a potential starter culture for the fermentation of high-salt foods
Source: BMC Genomics. 2018 Feb 13;19:136. doi: 10.1186/s12864-018-4532-1 (PMC5810056; doi:10.1186/s12864-018-4532-1)

**Fig. S2**. Genetic organization of the ribose operon in *S. equorum.*


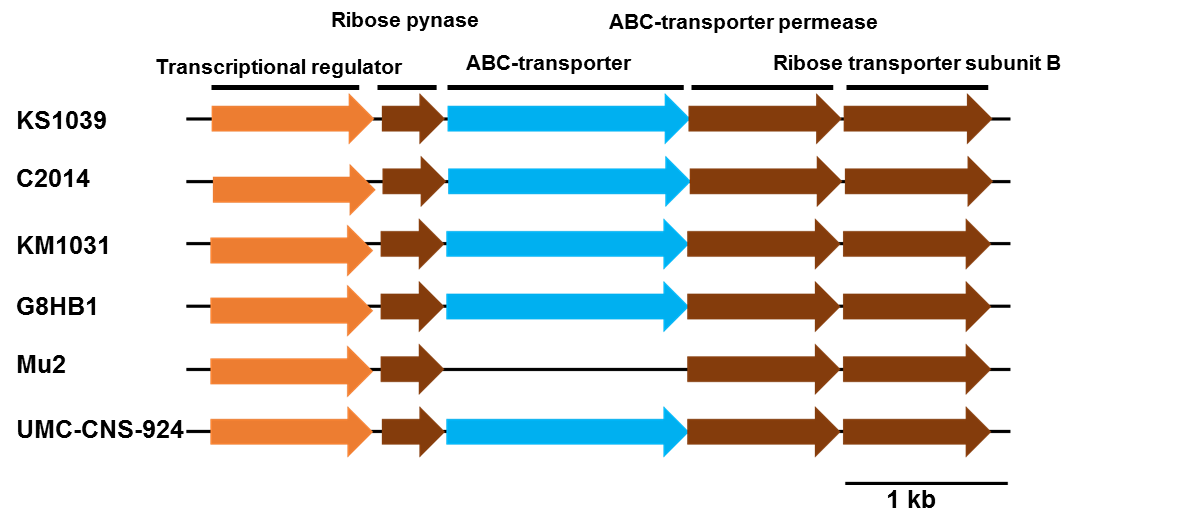

Supplement: Supplementary file 5 — Figure S2. Genetic organization of the ribose operon in S. equorum. (DOCX 70 kb) [file 12864_2018_4532_MOESM5_ESM.docx]

**Fig. S3**. Genetic organization of nitrogen metabolism in *S. equorum*.
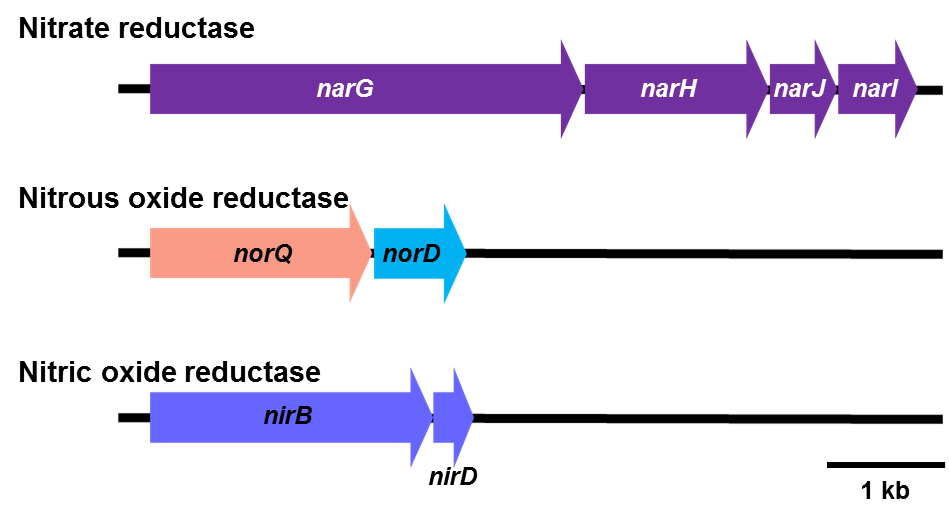

Supplement: Supplementary file 9 — Figure S3. Genetic organization of nitrogen metabolism in S. equorum. (DOCX 51 kb) [file 12864_2018_4532_MOESM9_ESM.docx]
