# Supplementary material for: Genomic insights into Staphylococcus equorum KS1039 as a potential starter culture for the fermentation of high-salt foods
Source: BMC Genomics. 2018 Feb 13;19:136. doi: 10.1186/s12864-018-4532-1 (PMC5810056; doi:10.1186/s12864-018-4532-1)
Supplement: Supplementary file 6 — Table S4. List of genes involved in amino acid biosynthesis. (DOCX 33 kb) [file 12864_2018_4532_MOESM6_ESM.docx]

Table S4. List of genes involved in amino acid biosynthesis.

| **Enzyme** | **Gene** | **KS1039** | **C2014** | **KM1031** | **G8HB1** | **Mu2** | **UMC-CNS-924** |
| --- | --- | --- | --- | --- | --- | --- | --- |
| Aspartokinase | *ak* | SE1039_RS05710 | AVJ22_RS05235 | AWC34_RS05350 | UF72_RS04140 | SEQMU2_RS10560 | SEQU_RS16830 |
| N-Acetylglutamate synthase | *argA* | SE1039_RS11845 | AVJ22_RS11730 | AWC34_RS11430 | UF72_RS10885 | SEQMU2_RS03690 | SEQU_RS17360 |
| Acetylglutamate kinase | *argB* | SE1039_RS11840 | AVJ22_RS11725 | AWC34_RS11425 | UF72_RS10880 | SEQMU2_RS03685 | SEQU_RS17355 |
| N-Acetyl-γ-glutamyl-phosphate reductase | *argC* | SE1039_RS11850 | AVJ22_RS11735 | AWC34_RS11435 | UF72_RS10890 | SEQMU2_RS03695 | SEQU_RS17365 |
| Acetylornithine aminotransferase | *argD* | SE1039_RS11855 | AVJ22_RS11740 | AWC34_RS11440 | UF72_RS10895 | SEQMU2_RS03700 | SEQU_RS17370 |
| Ornithine carbamoyltransferase | *argF* | SE1039_RS11805 | AVJ22_RS11680 | AWC34_RS11390 | UF72_RS10845 | SEQMU2_RS03650 | SEQU_RS17320 |
| Argininosuccinate synthase | *argG* | SE1039_RS03630 | AVJ22_RS03455 | AWC34_RS03665 | UF72_RS02160 | SEQMU2_RS08855 | SEQU_RS21935 |
| Argininosuccinate lyase | *argH* | SE1039_RS03625 | AVJ22_RS03450 | AWC34_RS03660 | UF72_RS02155 | SEQMU2_RS08850 | SEQU_RS21930 |
| Ornithine acetyltransferase/amino-acid acetyltransferase | *argJ* | SE1039_RS11845 | AVJ22_RS11730 | AWC34_RS11430 | UF72_RS10885 | SEQMU2_RS03690 | SEQU_RS17360 |
| 3-Phosphoshikimate 1-carboxyvinyltransferase | *aroA* | SE1039_RS06505 | AVJ22_RS06330 | AWC34_RS06075 | UF72_RS04850 | SEQMU2_RS11615 | SEQU_RS19875 |
| 3-Dehydroquinate synthase | *aroB* | SE1039_RS06510 | AVJ22_RS06335 | AWC34_RS06080 | UF72_RS04855 | SEQMU2_RS11620 | SEQU_RS19880 |
| Chorismate synthase | *aroC* | SE1039_RS06515 | AVJ22_RS06340 | AWC34_RS06085 | UF72_RS04860 | SEQMU2_RS11625 | SEQU_RS19885 |
| 3-Dehydroquinate dehydratase | *aroD* | SE1039_RS03320 | AVJ22_RS03150 | AWC34_RS03350 | UF72_RS01840 | SEQMU2_RS08530 | SEQU_RS25090 |
| Shikimate 5-dehydrogenase | *aroE* | SE1039_RS07110 | AVJ22_RS06955 | AWC34_RS06680 | UF72_RS05460 | SEQMU2_RS12220 | SEQU_RS19410 |
| Chorismate synthase | *aroF* | SE1039_RS04015 | AVJ22_RS03835 | AWC34_RS04010 | UF72_RS02505 | SEQMU2_RS09225 | SEQU_RS22280 |
| Phospho-2-dehydro-3-deoxyheptonate aldolase | *aroG* | SE1039_RS07765 | AVJ22_RS07620 | AWC34_RS07350 | UF72_RS06115 | SEQMU2_RS12890 | SEQU_RS18745 |
| Chorismate mutase | *aroH* | SE1039_RS07325 | AVJ22_RS07170 | AWC34_RS06900 | UF72_RS05670 | SEQMU2_RS12430 | SEQU_RS19195 |
| Biosynthetic Aromatic amino acid aminotransferase | *aroJ* | SE1039_RS02785 | AVJ22_RS02625 | AWC34_RS02835 | UF72_RS01295 | SEQMU2_RS07575 | SEQU_RS21595 |
| Aspartate-semialdehyde dehydrogenase | *asd* | SE1039_RS06135 | AVJ22_RS05975 | AWC34_RS05710 | UF72_RS04500 | SEQMU2_RS11185 | SEQU_RS19515 |
| Serine acetyltransferase | *cysE* | SE1039_RS01500 | AVJ22_RS01370 | AWC34_RS01390 | UF72_RS00060 | SEQMU2_RS00060 | SEQU_RS23800 |
| O-Acetyl-serine thiol-lyase A | *cysK* | SE1039_RS01340 | AVJ22_RS01210 | AWC34_RS01230 | UF72_RS10665 | SEQMU2_RS03470 | SEQU_RS17140 |
| Ketol-acid reductoisomerase | *ilvC* | SE1039_RS08890 | AVJ22_RS08805 | AWC34_RS08465 | UF72_RS13195 | SEQMU2_RS00780 | SEQU_RS26010 |
| Dihydroxy-acid dehydratase | *ilvD1* | SE1039_RS01050 | AVJ22_RS00920 | AWC34_RS00940 | UF72_RS06510 | SEQMU2_RS06635 | SEQU_RS23305 |
| Branched-chain amino acid aminotransferase | *ilvE* | SE1039_RS01825 | AVJ22_RS01690 | AWC34_RS01770 | UF72_RS00440 | SEQMU2_RS05145 | SEQU_RS23410 |
| Acetolactate synthase large subunit | *ilvG* | SE1039_RS08880 | AVJ22_RS08795 | AWC34_RS08455 | UF72_RS13185 | SEQMU2_RS00770 | SEQU_RS26020 |
| 3-Isopropylmalate dehydratase large subunit | *leuC* | SE1039_RS08905 | AVJ22_RS08815 | AWC34_RS08480 | UF72_RS13210 | SEQMU2_RS00795 | SEQU_RS25995 |
| 3-Isopropylmalate dehydratase small subunit | *leuD* | SE1039_RS08910 | AVJ22_RS08825 | AWC34_RS08485 | UF72_RS13215 | SEQMU2_RS00800 | SEQU_RS25990 |
| NADP-Specific glutamate dehydrogenase | *gdhA* | SE1039_RS01790 | AVJ22_RS01650 | AWC34_RS01735 | UF72_RS00405 | SEQMU2_RS05110 | SEQU_RS23445 |
| Glutamine synthetase | *glnS* | SE1039_RS05665 | AVJ22_RS05190 | AWC34_RS05305 | UF72_RS04095 | SEQMU2_RS10515 | SEQU_RS16785 |
| Ferredoxin-dependent glutamate synthase | *gltB2* | SE1039_RS10810 | AVJ22_RS10790 | AWC34_RS10385 | UF72_RS09845 | SEQMU2_RS02690 | SEQU_RS15080 |
| Glutamate synthase | *gltB* | SE1039_RS01095 | AVJ22_RS00965 | AWC34_RS00985 | UF72_RS06465 | SEQMU2_RS06680 | SEQU_RS23350 |
| Glutamate synthase | *gltD* | SE1039_RS01100 | AVJ22_RS00970 | AWC34_RS00990 | UF72_RS06460 | SEQMU2_RS06685 | SEQU_RS23355 |
| Serine hydroxymethyltransferase | *glyA* | SE1039_RS09160 | AVJ22_RS09085 | AWC34_RS08735 | UF72_RS11805 | SEQMU2_RS01035 | SEQU_RS24275 |
| ATP Phosphoribosyltransferase | *hisA* | SE1039_RS10850 | AVJ22_RS10830 | AWC34_RS10425 | UF72_RS09885 | SEQMU2_RS02730 | SEQU_RS15120 |
| Phosphoribosyl-ATP-pyrophosphatase | *hisB* | SE1039_RS10890 | AVJ22_RS10870 | AWC34_RS10465 | UF72_RS09925 | SEQMU2_RS02770 | SEQU_RS15160 |
| Phosphoribosylformimino-5-aminoimidazole carboxamide ribotide isomerase | *hisD* | SE1039_RS10880 | AVJ22_RS10860 | AWC34_RS10455 | UF72_RS09915 | SEQMU2_RS02760 | SEQU_RS15150 |
| Imidazole glycerol phosphate synthase cyclase subunit | *hisEa* | SE1039_RS10885 | AVJ22_RS10865 | AWC34_RS10460 | UF72_RS09920 | SEQMU2_RS02765 | SEQU_RS15155 |
| Imidazole glycerol phosphate synthase amidotransferase subunit | *hisEb* | SE1039_RS10875 | AVJ22_RS10855 | AWC34_RS10450 | UF72_RS09910 | SEQMU2_RS02755 | SEQU_RS15145 |
| Imidazoleglycerol-phosphate dehydratase | *hisF* | SE1039_RS10870 | AVJ22_RS10850 | AWC34_RS10445 | UF72_RS09905 | SEQMU2_RS02750 | SEQU_RS15140 |
| Histidinol-phosphate aminotransferase | *hisG* | SE1039_RS02785 | AVJ22_RS02625 | AWC34_RS02835 | UF72_RS01295 | SEQMU2_RS07575 | SEQU_RS21595 |
| Histidinol dehydrogenase | *hisI* | SE1039_RS12405 | AVJ22_RS12325 | AWC34_RS11975 | UF72_RS08320 | SEQMU2_RS02740 | SEQU_RS15130 |
| Homoserine dehydrogenase | *hom* | SE1039_RS05715 | AVJ22_RS05240 | AWC34_RS05355 | UF72_RS04145 | SEQMU2_RS10565 | SEQU_RS16835 |
| Histidine ammonia-lyase | *hutH* | SE1039_RS13475 | AVJ22_RS13235 | AWC34_RS12770 | UF72_RS07475 | SEQMU2_RS05560 | SEQU_RS20825 |
| Urocanate hydratase | *hutU* | SE1039_RS10185 | AVJ22_RS10165 | AWC34_RS09760 | UF72_RS09215 | SEQMU2_RS02045 | SEQU_RS14450 |
| Imidazolonepropionase | *hutI* | SE1039_RS10180 | AVJ22_RS10160 | AWC34_RS09755 | UF72_RS09210 | SEQMU2_RS02040 | SEQU_RS14445 |
| Formiminoglutamase | *hutG* | SE1039_RS10200 | AVJ22_RS10180 | AWC34_RS09775 | UF72_RS09230 | SEQMU2_RS02060 | SEQU_RS14465 |
| 5-Methyl tetrahydropteroyltriglutamate/homocysteine methyltransferase | *metE* | SE1039_RS00365 | AVJ22_RS00240 | AWC34_RS00280 | UF72_RS07160 | SEQMU2_RS05870 | SEQU_RS20515 |
| Prephenate dehydratase | *pheA* | SE1039_RS08650 | AVJ22_RS08525 | AWC34_RS08230 | UF72_RS12955 | SEQMU2_RS00525 | SEQU_RS24835 |
| Ornithine aminotransferase | *rocD* | SE1039_RS11855 | AVJ22_RS11740 | AWC34_RS11440 | UF72_RS10895 | SEQMU2_RS03700 | SEQU_RS17370 |
| Arginase | *rocF* | SE1039_RS09420 | AVJ22_RS09345 | AWC34_RS08995 | UF72_RS12065 | SEQMU2_RS01295 | SEQU_RS24535 |
| Phosphoglycerate dehydrogenase | *serA* | SE1039_RS05995 |  | AWC34_RS05580 | UF72_RS04370 | SEQMU2_RS11060 | SEQU_RS17060 |
|  |  | SE1039_RS07705 | AVJ22_RS07560 | AWC34_RS07290 | UF72_RS06055 | SEQMU2_RS12820 | SEQU_RS18805 |
|  |  | SE1039_RS08405 | AVJ22_RS08275 | AWC34_RS07985 | UF72_RS12710 | SEQMU2_RS13515 | SEQU_RS24590 |
| Phosphoserine phosphatase | *serB* | SE1039_RS07710 | AVJ22_RS07565 | AWC34_RS07295 | UF72_RS06060 | SEQMU2_RS12825 | SEQU_RS18800 |
| Phosphoserine aminotransferase | *serC* | SE1039_RS07700 | AVJ22_RS07555 | AWC34_RS07285 | UF72_RS06050 | SEQMU2_RS12815 | SEQU_RS18810 |
| Anthranilate synthase | *thrA* | SE1039_RS06025 | AVJ22_RS05865 | AWC34_RS05600 | UF72_RS04390 | SEQMU2_RS11075 | SEQU_RS17080 |
|  |  | SE1039_RS06030 | AVJ22_RS05870 | AWC34_RS05605 | UF72_RS04395 | SEQMU2_RS11080 | SEQU_RS17085 |
| Homoserine kinase | *thrB* | SE1039_RS05725 | AVJ22_RS05250 | AWC34_RS05365 | UF72_RS04155 | SEQMU2_RS10575 | SEQU_RS16845 |
| Threonine synthase | *thrC* | SE1039_RS05720 | AVJ22_RS05245 | AWC34_RS05360 | UF72_RS04150 | SEQMU2_RS10570 | SEQU_RS16840 |
| Tryptophan synthase, alpha subunit | *trpA* | SE1039_RS06055 | AVJ22_RS05895 | AWC34_RS05630 | UF72_RS04420 | SEQMU2_RS11105 | SEQU_RS17110 |
| Tryptophan synthase, β subunit | *trpB* | SE1039_RS06050 | AVJ22_RS05890 | AWC34_RS05625 | UF72_RS04415 | SEQMU2_RS11100 | SEQU_RS17105 |
| Indole-3-glycerol phosphate synthase | *trpC* | SE1039_RS06040 | AVJ22_RS05880 | AWC34_RS05615 | UF72_RS04405 | SEQMU2_RS11090 | SEQU_RS17095 |
| Anthranilate phosphoribosyltransferase | *trpD* | SE1039_RS06035 | AVJ22_RS05875 | AWC34_RS05610 | UF72_RS04400 | SEQMU2_RS11085 | SEQU_RS17090 |
| Prephenate dehydrogenase | *tyrA* | SE1039_RS05990 | AVJ22_RS05810 | AWC34_RS05575 | UF72_RS04365 | SEQMU2_RS11055 | SEQU_RS17055 |
| N-Acetyl-L,L-diaminopimelate aminotransferase | ADAPAT | SE1039_RS04040 | AVJ22_RS03860 | AWC34_RS04035 | UF72_RS02530 | SEQMU2_RS09250 | SEQU_RS22305 |
| 2-Amino-3-ketobutyrate coenzyme A ligase | AKBL | SE1039_RS01620 | AVJ22_RS01475 | AWC34_RS01495 | UF72_RS00165 | SEQMU2_RS00165 | SEQU_RS23695 |
| Branched-chain amino acid aminotransferase | BCAT | SE1039_RS01825 | AVJ22_RS01690 | AWC34_RS01770 | UF72_RS00440 | SEQMU2_RS05145 | SEQU_RS23410 |
| Branched-chain alpha-keto acid dehydrogenase | BCDH_E1 | SE1039_RS04275 | AVJ22_RS04100 | AWC34_RS04270 | UF72_RS02765 | SEQMU2_RS09485 | SEQU_RS15755 |
| Dihydrolipoamide acyltransferase component of branched-chain alpha-keto acid dehydrogenase complex | BCDH_E2b | SE1039_RS06705 | AVJ22_RS06530 | AWC34_RS06275 | UF72_RS05050 | SEQMU2_RS11815 | SEQU_RS20075 |
| Dihydrolipoamide dehydrogenase of branched-chain alpha-keto acid dehydrogenase | BCDH_E3 | SE1039_RS06720 | AVJ22_RS06545 | AWC34_RS06290 | UF72_RS05065 | SEQMU2_RS11830 | SEQU_RS20090 |
| Cystathionine gamma-lyase | CTGL | SE1039_RS11610 | AVJ22_RS11540 | AWC34_RS11205 | UF72_RS10660 | SEQMU2_RS03465 | SEQU_RS17135 |
| Cystathionine beta-lyase | CTBL | SE1039_RS00375 | AVJ22_RS00250 | AWC34_RS00290 | UF72_RS07150 | SEQMU2_RS05880 | SEQU_RS20505 |
| N-Acetyl-L,L-diaminopimelate deacetylase | DAPDA | SE1039_RS01615 | AVJ22_RS01470 | AWC34_RS01490 | UF72_RS00160 | SEQMU2_RS00160 | SEQU_RS23700 |
| Diaminopimelate epimerase | DAPE | SE1039_RS06160 | AVJ22_RS06000 | AWC34_RS05735 | UF72_RS04525 | SEQMU2_RS11210 | SEQU_RS19540 |
| Diaminopimelate decarboxylase | DAPDC | SE1039_RS06165 | AVJ22_RS06005 | AWC34_RS05740 | UF72_RS04530 | SEQMU2_RS11215 | SEQU_RS19545 |
| 4-Hydroxy-tetrahydrodipicolinate synthase | DHPS | SE1039_RS11755 | AVJ22_RS11650 | AWC34_RS11350 | UF72_RS10805 | SEQMU2_RS03610 | SEQU_RS17280 |
|  |  | SE1039_RS11095 | AVJ22_RS11065 | AWC34_RS10670 | UF72_RS10130 | SEQMU2_RS02975 | SEQU_RS15365 |
| 4-Hydroxy-tetrahydrodipicolinate reductase | DHPR | SE1039_RS03780 | AVJ22_RS03615 | AWC34_RS03820 | UF72_RS02315 | SEQMU2_RS09010 | SEQU_RS22090 |
|  |  | SE1039_RS06145 | AVJ22_RS05985 | AWC34_RS05720 | UF72_RS04510 | SEQMU2_RS11195 | SEQU_RS19525 |
| Homocysteine S-methyltransferase | HSM | SE1039_RS01800 | AVJ22_RS01660 | AWC34_RS01745 | UF72_RS00415 | SEQMU2_RS05120 | SEQU_RS23435 |
| S-Adenosylmethionine synthetase | SAMS | SE1039_RS07995 | AVJ22_RS07860 | AWC34_RS07580 | UF72_RS06360 | SEQMU2_RS13120 | SEQU_RS18515 |
| L-Threonine 3-dehydrogenase | TDG_9 | SE1039_RS01820 | AVJ22_RS01685 | AWC34_RS01765 | UF72_RS00435 | SEQMU2_RS05140 | SEQU_RS23415 |
| 2,3,4,5-Tetrahydropyridine-2,6-dicarboxylate N-acetyltransferase | THPAT | SE1039_RS06150 | AVJ22_RS05990 | AWC34_RS05725 | UF72_RS04515 | SEQMU2_RS11200 | SEQU_RS19530 |
