# Supplementary material for: Genomic insights into Staphylococcus equorum KS1039 as a potential starter culture for the fermentation of high-salt foods
Source: BMC Genomics. 2018 Feb 13;19:136. doi: 10.1186/s12864-018-4532-1 (PMC5810056; doi:10.1186/s12864-018-4532-1)
Supplement: Supplementary file 7 — Table S5. List of genes coding for proteases and peptidases. (DOCX 20 kb) [file 12864_2018_4532_MOESM7_ESM.docx]

Table S5. List of genes coding for proteases and peptidases.

| **enzyme** | **KS1039** | **C2014** | **KM1031** | **G8HB1** | **Mu2** | **UMC-CNS-924** |
| --- | --- | --- | --- | --- | --- | --- |
| Serine protease | SE1039_RS03885 | AVJ22_RS03720 | AWC34_RS03925 | UF72_RS02420 |  | SEQU_RS22195 |
|  | SE1039_RS06315 | AVJ22_RS06120 | AWC34_RS05880 | UF72_RS04660 | SEQMU2_RS11425 | SEQU_RS19685 |
|  | SE1039_RS07010 | AVJ22_RS06835 | AWC34_RS06580 | UF72_RS05360 | SEQMU2_RS12120 | SEQU_RS20370 |
|  | SE1039_RS07725 | AVJ22_RS07580 | AWC34_RS07310 | UF72_RS06075 | SEQMU2_RS12850 | SEQU_RS18785 |
| CAAX amino terminal protease | SE1039_RS03150 | AVJ22_RS02970 | AWC34_RS03175 | UF72_RS01665 | SEQMU2_RS08360 | SEQU_RS25280 |
|  | SE1039_RS03900 | AVJ22_RS03735 | AWC34_RS03940 | UF72_RS02435 | SEQMU2_RS09130 | SEQU_RS22210 |
|  | SE1039_RS08770 | AVJ22_RS08690 | AWC34_RS08350 | UF72_RS13075 | SEQMU2_RS00660 | SEQU_RS26130 |
|  | SE1039_RS10805 | AVJ22_RS10785 | AWC34_RS10380 | UF72_RS09840 | SEQMU2_RS02685 | SEQU_RS15075 |
| Aminopeptidease | SE1039_RS03520 | AVJ22_RS03355 | AWC34_RS03555 | UF72_RS02050 | SEQMU2_RS08745 | SEQU_RS24885 |
|  | SE1039_RS06015 | AVJ22_RS08395 | AWC34_RS08105 | UF72_RS04380 | SEQMU2_RS11065 | SEQU_RS17070 |
|  | SE1039_RS07805 | AVJ22_RS10800 | AWC34_RS10395 | UF72_RS06155 | SEQMU2_RS12930 | SEQU_RS18705 |
|  | SE1039_RS10820 | AVJ22_RS10800 | AWC34_RS10395 | UF72_RS09855 | SEQMU2_RS02700 | SEQU_RS15090 |
|  | SE1039_RS08480 | AVJ22_RS08350 | AWC34_RS08060 | UF72_RS12785 | SEQMU2_RS00355 | SEQU_RS24665 |
|  | SE1039_RS08525 | AVJ22_RS08395 | AWC34_RS08105 | UF72_RS12830 | SEQMU2_RS00400 | SEQU_RS24710 |
|  | SE1039_RS10685 | AVJ22_RS10670 | AWC34_RS10260 | UF72_RS09720 | SEQMU2_RS02565 | SEQU_RS14955 |
| Oligoendopeptidase | SE1039_RS03775 | AVJ22_RS03610 | AWC34_RS03815 | UF72_RS02310 | SEQMU2_RS09005 | SEQU_RS22085 |
|  | SE1039_RS06085 | AVJ22_RS05925 | AWC34_RS05660 | UF72_RS04450 | SEQMU2_RS11135 | SEQU_RS19465 |
| Proline peptidase | SE1039_RS06785 | AVJ22_RS06610 | AWC34_RS06350 | UF72_RS05130 | SEQMU2_RS11895 | SEQU_RS20155 |
|  | SE1039_RS07605 | AVJ22_RS06310 | AWC34_RS06055 | UF72_RS05955 | SEQMU2_RS12720 | SEQU_RS18905 |
|  | SE1039_RS08005 | AVJ22_RS07870 | AWC34_RS07590 | UF72_RS06370 | SEQMU2_RS13130 | SEQU_RS18505 |
