# Supplementary material for: Genomic insights into Staphylococcus equorum KS1039 as a potential starter culture for the fermentation of high-salt foods
Source: BMC Genomics. 2018 Feb 13;19:136. doi: 10.1186/s12864-018-4532-1 (PMC5810056; doi:10.1186/s12864-018-4532-1)
Supplement: Supplementary file 8 — Table S6. List of the genes involved in protein and peptide transport systems. (DOCX 19 kb) [file 12864_2018_4532_MOESM8_ESM.docx]

Table S6. List of the genes involved in protein and peptide transport systems.

|  | Protein | KS1039 | C2014 | KM1031 | G8HB1 | Mu2 | UMC-CNS-924 |
| --- | --- | --- | --- | --- | --- | --- | --- |
| Oligopeptide ABC transport system | OppA | SE1039_RS11550 | AVJ22_RS11480 | AWC34_RS11145 | UF72_RS10600 | SEQMU2_RS03405 | SEQU_RS24145 |
|  | OppA | SE1039_RS03750 | AVJ22_RS03585 | AWC34_RS03790 | UF72_RS02285 | SEQMU2_RS08980 | SEQU_RS22060 |
|  | OppB | SE1039_RS03730 | AVJ22_RS03565 | AWC34_RS03770 | UF72_RS02265 | SEQMU2_RS08960 | SEQU_RS22040 |
|  | OppC | SE1039_RS03735 | AVJ22_RS03570 | AWC34_RS03775 | UF72_RS02270 | SEQMU2_RS08965 | SEQU_RS22045 |
|  | OppD | SE1039_RS03740 | AVJ22_RS03575 | AWC34_RS03780 | UF72_RS02275 | SEQMU2_RS08970 | SEQU_RS22050 |
|  | OppF | SE1039_RS03745 | AVJ22_RS03580 | AWC34_RS03785 | UF72_RS02280 | SEQMU2_RS08975 | SEQU_RS22055 |
| ABC transporter | EcsA | SE1039_RS08115 | AVJ22_RS07975 | AWC34_RS07700 | UF72_RS13485 | SEQMU2_RS13240 | SEQU_RS18395 |
|  | EcsB | SE1039_RS08120 | AVJ22_RS07980 | AWC34_RS07705 | UF72_RS13480 | SEQMU2_RS13245 | SEQU_RS18390 |
|  | DppD | SE1039_RS11530 | AVJ22_RS11460 | AWC34_RS11120 | UF72_RS10580 | SEQMU2_RS03385 | SEQU_RS24125 |
|  | DppC | SE1039_RS11540 | AVJ22_RS11470 | AWC34_RS11135 | UF72_RS10590 | SEQMU2_RS03395 | SEQU_RS24135 |
| Peptide ABC transporter permease | YjdL | SE1039_RS02800 | AVJ22_RS02640 | AWC34_RS02850 | UF72_RS01310 | SEQMU2_RS07590 | SEQU_RS21615 |
|  | YjdL | SE1039_RS02805 | AVJ22_RS02645 | AWC34_RS02855 | UF72_RS01315 | SEQMU2_RS07595 | SEQU_RS21615 |
