# Supplementary material for: Genomic insights into Staphylococcus equorum KS1039 as a potential starter culture for the fermentation of high-salt foods
Source: BMC Genomics. 2018 Feb 13;19:136. doi: 10.1186/s12864-018-4532-1 (PMC5810056; doi:10.1186/s12864-018-4532-1)
Supplement: Supplementary file 10 — Figure S4. Growth inhibition of S. aureus by S. equorum strains. S. aureus RN4220 was used as an indicator strain. (DOCX 336 kb) [file 12864_2018_4532_MOESM10_ESM.docx]

**Fig. S4.** Growth inhibition of *S. aureus* by *S. equorum* strains. *S. aureus* RN4220 was used as an indicator strain.

**
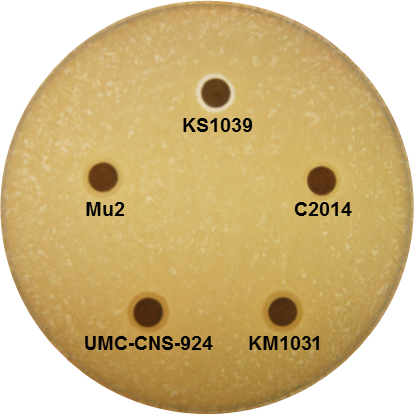
**
